# Editorial: Kv7 Channels: Structure, Physiology, and Pharmacology

**DOI:** 10.3389/fphys.2021.679317

**Published:** 2021-04-16

**Authors:** Thomas A. Jepps, Vincenzo Barrese, Francesco Miceli

**Affiliations:** ^1^Vascular Biology Group, Department of Biomedical Sciences, University of Copenhagen, Copenhagen, Denmark; ^2^Department of Neuroscience, Reproductive Sciences and Dentistry, University of Naples Federico II, Naples, Italy

**Keywords:** Kv7 channels, KCNQ channel, physiology, pharmacology, ion channels

The Kv7 family of voltage-gated potassium (K^+^) channels consists of five members (Kv7.1-7.5) encoded for by the KCNQ1-5 genes, respectively. Kv7 channels assemble as tetramers of identical or compatible α subunits, with each α subunit consisting of six transmembrane segments (S1–S6) and cytoplasmic N- and C-termini. The transmembrane segments between S1 and S4 form the voltage-sensing domain (VSD), where the S4 segment is crucial for channel gating as it contains positively charged arginines (Rs), each separated by non-polar residues. The S5 and S6 segments and the interconnecting loop participate to the formation of the pore domain (PD). Kv7 channels are characterized by a long intracellular C-terminus, containing domains necessary for tetramerization and involved in binding and transductional activity by critical regulators, such as phosphatidylinositol 4,5-bisphosphate (PIP2), calmodulin (CaM), syntaxin, A-kinase-anchoring proteins and proteinkinase C, and ankyrin-G (Barrese et al., 2018).

Kv7 channels produce currents that are voltage-activated, slowly activating and non-inactivating, which were first recorded in bullfrog sympathetic neurons (Brown and Adams, 1980). At this time, the molecular identity of the channels was unknown, so the K^+^ current was termed the “M-current,” which derives from the observation that muscarinic acetylcholine receptor agonist, muscarine, inhibited this voltage-sensitive K^+^ current (Brown and Adams, 1980). The muscarinic acetylcholine receptors are coupled to Gq proteins, and it is now known that activation of other Gq protein-coupled receptors will also inhibit the M-current by depleting the levels of PIP_2_ in the membrane (Marrion, 1997; Suh and Hille, 2002; Ford et al., 2003; Zhang et al., 2003). Kv7 channels, unlike other Kv channels, are highly sensitive to PIP_2_, which acts as a cofactor that mediates coupling of the VSD with the PD. Thus, without PIP_2_, membrane depolarization does not cause pore opening (Delmas and Brown, 2005; Zaydman et al., 2013).

Several years later, Kv7.1 (KCNQ1) expression was first identified in the heart (Barhanin et al., 1996; Sanguinetti et al., 1996), where it is responsible for the slowly activating delayed rectifier K^+^ current “I_Ks_.” Mutations in KCNQ1 (or its ancillary subunit, KCNE1) cause a large number of hereditary arrhythmias that are manifest as a prolongation of the QT interval in electrocardiograms (LQT1 syndrome: Wang et al., 1996; Herbert et al., 2002). In 1998, KCNQ2 and KCNQ3 (encoding Kv7.2 and Kv7.3 proteins, respectively) were cloned, and mutations in these genes were associated to a form of epilepsy called Benign Familial Neonatal Convulsions (BFNC: Biervert et al., 1998; Charlier et al., 1998; Singh et al., 1998). Wang et al. (1998) demonstrated that the kinetic properties of the Kv7.2/7.3 heteromeric channel resembled closely those of the native M-current, which was also inhibited by muscarine. Later, Kv7.4 (KCNQ4) was identified on the basal membrane of the outer hair cells of the inner ear and auditory nerves, with mutations to the channel associated with autosomal dominant deafness (DFNA2; Kubisch et al., 1999; Kharkovets et al., 2000, 2006; Søgaard et al., 2001). Finally, Kv7.5 (KCNQ5) was identified as a molecular correlate of the M-current by forming heteromultimers with Kv7.3 (Lerche et al., 2000; Schroeder et al., 2000; Shah et al., 2002).

Many cell types throughout the body depend functionally on Kv7 channel expression, with mutations to these channels, or their ancillary subunits, underling multiple diseases. Research interest in these channels continues to grow as their involvement in disease and potential as therapeutic targets becomes more apparent (Figure 1).

**Figure 1:**
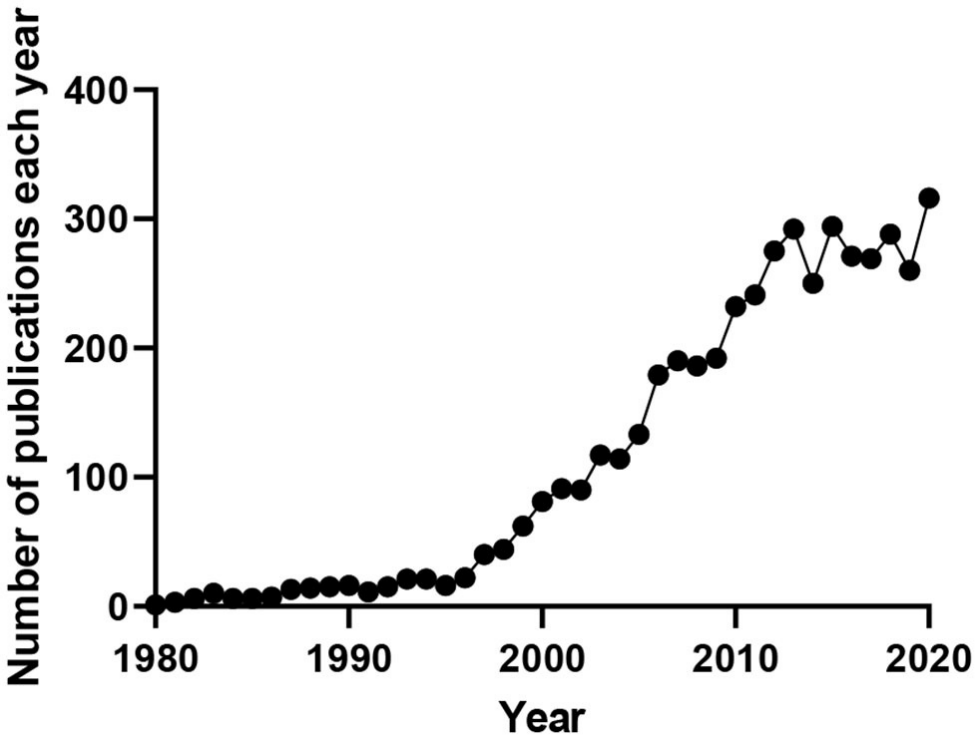
Number of publications produced per year containing the terms “M-current,” “KCNQ(X),” or “Kv7(.X).” Data obtained from www.pubmed.ncbi.nlm.nih.gov.

With such a large and continuously growing interest in Kv7 channel research, a special Research Topic in Frontiers in Physiology was created following the International Kv7 Channels Symposium in Naples, Italy, 2019. This Research Topic has collected 17 articles, including seven original research papers, three mini-reviews, and seven full reviews from prominent scientists in the field. The collection of papers in this Research Topic expands the current knowledge on the structure, pathophysiology, and pharmacology of Kv7 channels.

## Kv7 Channel Regulation

Kv7 channels are voltage-gated channels and Wang et al. summarize the most recent knowledge on Kv7.1 assembly, kinetics of activation and inactivation, voltage-sensor pore coupling, and regulation by KCNE subunits as well as other signaling molecules. As well as being voltage-dependent, several endogenous small molecules can modulate Kv7 channel activity. The study by Greenwood and Stott builds on previous work showing G-protein βγ subunits are obligatory for Kv7.4 channel activity. In particular, Gβ3 was shown to be necessary for Kv7 activity in rat renal vascular smooth muscle cells. Larsson et al. review the regulation of Kv7 channels by Polyunsaturated Fatty Acids (PUFAs). In particular, PUFAs increase the current and produce a leftward shift in the voltage-dependence through both direct and indirect mechanisms. CaM is often regarded an essential auxiliary subunit of Kv7 channels that modulates function. The work by Zhuang and Yan identifies structural determinants that are necessary for CaM and PIP_2_ regulation. Their work shows that the third EF hand (EF3) of CaM is required for the calcium-dependent regulation of Kv7.4 activation, and that the S2–S3 loop of Kv7.4 is essential for the regulation mediated by CaM. Tran et al. described two variants of the CaM-binding Helix A of Kv7.2 channels in two patients with developmental and epileptic encephalopathy (DEE). Functional studies revealed that these two variants suppressed channel current density and impaired CaM and PIP_2_ regulation, a result consistent with the severe DEE phenotype associated with these mutations. Interestingly, the novel Kv7.2/7.3 channel-selective opener, SF0034, rescued current amplitudes of the mutated channels suggesting a potential therapy for KCNQ2 encephalopathy.

Abbott reviews our current perception of Kv7 channels as simply voltage-dependent channels, questioning whether they should be considered ligand-gated channels that are also sensitive to voltage in certain cell types. The review presents our current knowledge on the regulation of Kv7 channels by voltage, KCNE subunits, PIP_2_, and CaM, as well as neurotransmitters such as GABA and GABA-related endogenous metabolites β-hydroxybutyric acid (BHB) and γ-amino-β-hydroxybutyric acid (GABOB). Van der Horst et al. discuss another voltage-independent regulatory mechanism in their review focusing on the complex regulation of Kv7 channels following cAMP-activation in multiple cell types.

## Pharmacological Targeting of Kv7 Channels in Neurological Diseases

Hoshi discusses the molecular mechanisms controlling suppression of the M-current in neurones, the relevance of lipid metabolism as well as the potential therapeutic implications in epilepsy and contribution of these mechanisms to the anti-seizure effect of valproic acid. These findings, along with those highlighted in the Abbott review about the direct binding of BHB to Kv7.3 channels, validate the therapeutic potential of Kv7 channel activation in epilepsy, as also demonstrated by the Miceli study. Miceli et al. describe the first missense loss-of-function (LoF) pathogenic variant within the S4 segment of Kv7.3, identified in three individuals with BFNE. Functional studies revealed that homomeric Kv7.3 M240R channels were not functional, whereas heteromeric channels incorporating wild type Kv7.2, wild type Kv7.3, and Kv7.3 M240R subunits (at cDNA ratio of 1:0.5:0.5, respectively), a stoichiometry reproducing the genetic combination occurring in the affected family members, displayed a depolarizing shift in activation gating. Interestingly, the functional deficit caused by both mutations could be restored partially by the pharmacological activation of Kv7 current. In particular, the application of BHB, a ketone body generated during the ketogenic diet (KD), reversed channel dysfunction induced by the mutation in Kv7.3, thus providing the rationale for the use of KD in patients carrying LoF variants in Kv7.2 or Kv7.3.

Mutations in KCNQ2 and KCNQ3 have more recently been associated with DEE (Weckhuysen et al., 2012). The clinical heterogeneity described in patients carrying mutations in Kv7.2 and Kv7.3 cannot be explained only with the severity of channel dysfunction, as the degree of developmental delay does not completely correlate with the frequency or severity of seizures. The review by Dirkx et al. discusses that Kv7 channels might have a crucial role beyond the control of neuronal firing. In particular, the differential expression and function of Kv7 channels during development might regulate fundamental steps such as differentiation, proliferation, and synaptogenesis that, if altered, profoundly modify brain structure and function. However, the heterogenous phenotype found in many epileptic patients might be explained with the close relationship and reciprocal influence between neuronal excitability, structural alterations, and neuronal plasticity. Baculis et al. review the current knowledge regarding the activity-dependent regulation of Kv7 channels at the synaptic level, and their contribution to intrinsic and synaptic plasticity. Such evidence also provides the molecular basis for the role of Kv7 channels in cognition and behavior, and further interesting insight into the association of KCNQ2 and KCNQ3 variants with developmental disorders and intellectual disability.

Robust evidence generated in the past two decades have convincingly expanded the spectrum of pathophysiological roles of Kv7 channels beyond epilepsy. These data have been the basis for novel therapeutic approaches for the treatment of several neurological diseases. Vigil et al. review the studies investigating the efficacy of Kv7 modulators in stroke, traumatic brain injury, drug addiction and mood disorders, and highlight the pharmacodynamic and pharmacokinetic issues (such as subunit selectivity and brain-specific delivery) that should be addressed to obtain more effective therapeutic treatments for these diseases.

## Kv7 Channels Can No Longer Be Regarded as “Cardiac” and “Neuronal” Channels

Since they were first discovered in neurones and cardiac myocytes, Kv7 channels are often termed the “cardiac” Kv7 channel (Kv7.1) and the “neuronal” Kv7 channels (Kv7.2-7.5). However, Kv7 channels are now recognized to play important physiological roles in many cell types, highlighted by several papers in this Research Topic. Kv7 channels are key determinants of vascular smooth muscle contractility. The study by Brueggemann et al. suggests a Kv7.4:Kv7.5 stoichiometry of 2:2, with alternating Kv7.4 and Kv7.5 α-subunits as the predominating channel in rat mesenteric artery smooth muscle cells. As mentioned previously, the study by Greenwood and Stott investigates the βγ subunit regulation of Kv7 channels in rat renal arteries. Ma et al. investigate the interactive regulation of the Kv7 and large-conductance Ca^2+^-sensitive K^+^ (BK) channels in rat arteries, showing that in adult arteries the functional impact of Kv7 channels increases after blocking BK channels. This study also showed aged-related changes in Kv7 channel activity, with blockade of BK channels having no effect on Kv7 channel activity in arteries from young rats. Keeping within the vascular wall, Baldwin et al. provide the first evidence of Kv7.4 and Kv7.5 channels within rat mesenteric endothelial cells and found they were involved in nitric oxide release in these vessels. Kv7 channels are expressed in a variety of non-vascular smooth muscle, and the review by Malysz and Petkov highlights the expression and functional role of Kv7 channels in urinary bladder smooth muscle. Mondejar-Parreño et al. review the impact of Kv7 channels on multiple aspects of pulmonary physiology. In their review, the role of Kv7 channels in several pulmonary diseases is discussed, as well as the therapeutic potential of targeting these channels.

## Conclusion

This Research Topic underscores the important physiological roles and pharmacological potential of Kv7 channels in multiple tissues. The papers in this Research Topic highlight the complexities of the Kv7 voltage- and ligand-gated potassium channels, and that there is still much to discover about the myriad of cell-dependent physiological roles for these channels.

## Author Contributions

All authors listed have made a substantial, direct and intellectual contribution to the work, and approved it for publication.

## Conflict of Interest

The authors declare that the research was conducted in the absence of any commercial or financial relationships that could be construed as a potential conflict of interest.
